# Therapeutic Effects of Saireito (Chai-Ling-Tang), a Traditional Japanese Herbal Medicine, on Lymphedema Caused by Radiotherapy: A Case Series Study

**DOI:** 10.1155/2013/241629

**Published:** 2013-06-04

**Authors:** Aiko Nagai, Yuta Shibamoto, Keiko Ogawa

**Affiliations:** ^1^Department of Therapeutic Radiology, Ishikawa Prefectural Central Hospital, Kanazawa, Ishikawa 920-8530, Japan; ^2^Department of Radiology, Nagoya City University, Graduate School of Medical Sciences, 1 Kawasumi, Mizuho-cho, Mizuho-ku, Nagoya, Aichi 467-8601, Japan; ^3^Clinic of Japanese-Oriental (Kampo) Medicine, Department of Otorhinolaryngology-Head and Neck Surgery, Kanazawa University Hospital, Kanazawa, Ishikawa 920-8641, Japan

## Abstract

Despite the development of radiotherapy machines and technologies, a proportion of patients suffer from radiation-induced lymphedema. Saireito (SRT) is a traditional Japanese herbal medicine that has been used for treating edema and inflammation in conditions such as nephritic disease. This study investigated the effect of SRT on lymphedema caused by radiotherapy. Four patients were treated with SRT at a dose of 9 g/day. The severity of lymphedema was evaluated using the Common Terminology Criteria for Adverse Events version 4 and Numerical Rating Scale before and after SRT treatment. After the treatment with SRT, 2 of 4 patients (50%) showed apparent improvement in lymphedema. One of the cases had difficulty in wearing the custom-made thermoplastic cast, but after SRT administration, he could wear the mask easily. One case decided to stop taking SRT 3 days after initiation because cough and fever appeared. In conclusion, it is important to control the side effects of radiotherapy, which leads to improved tumor control rates. Prospective randomized studies are necessary to confirm the findings of this case series study.

## 1. Introduction

 With the aging of the population, radiotherapy is taking an increasingly important role for preserving organ function. With the development of machines and technologies for radiotherapy, adverse effects have decreased. However, some patients still suffer from irreversible adverse events associated with radiotherapy. One of the most serious complications is lymphedema, which is a chronic, debilitating, refractory, or incurable condition. No consensus has been reached on the standard therapy for lymphedema. It is difficult to control lymphedema in Western medicine, and the long-term use of the applied medications has been limited due to documented hepatotoxicity [1–3]. The only effective types of primary care for lymphedema have been reported to be elastic clothing, multilayer lymphedema bandaging (MLLB), lymph drainage, skin care, and complex physical therapy. Complex physical therapy includes elastic clothing, MLLB, lymph drainage, skin care, and exercise [4–8]. However, the problem is that elastic clothing or MLLB cannot be used on the head and neck, so it is difficult to control lymphedema in these areas.

 Kampo, Japanese traditional herbal medicine, is the most frequently used type of alternative and complementary medicine in Japan. The aim of Kampo therapy is to improve patients' condition whatever their disease is. Kampo medicine is playing an increasingly important role in closing the gap between modern Western medicine and the demands of patients. Kampo applications for cancer therapy were developed by trial and error because of dissatisfaction with Western medicine in dealing with problems such as the adverse effects of radiotherapy, chemotherapy, and various types of general malaise [9]. For example, Daikenchuto is effective on postoperative adhesive small bowel obstruction requiring long-tube decompression [10] and Goshajinkigan has been used for peripheral neurotoxicity of oxaliplatin in patients with advanced or recurrent colorectal cancer [11]. The Japanese herbal medicine Saireito (SRT) has been used empirically in the treatment of various edematous disorders (nephritic syndrome, cirrhosis, pregnancy, swelling, and lymphedema after surgery and macular edema) [12, 13]. It is composed of 12 crude drugs in fixed proportions: 7.0 g of *Bupleurum root*, 5.0 g of *Pinellia tuber,* 5.0 g of *Alisma rhizome,* 3.0 g of *Scutellaria root,* 3.0 g of *ginseng,* 3.0 g of *Poria sclerotium,* 3.0 g of *Polypoms sclerotium,* 3.0 g of *Astractylodes lancea rhizome,* 3.0 g of *jujube,* 2.0 g of *Glycyrrhiza*, 2.0 g of *cinnamon bark,* and 1.0 g of *ginger.* Saikosaponin, which is derived from the medicinal plant *Bupleurum root,* exhibits a variety of pharmacological and immune-modulatory activities including anti-inflammatory responses [14]. In light of the purported activities of SRT for various edematous disorders in experimental models, we investigated whether SRT had beneficial effects in terms of the reduction of lymphedema in patients after or during radiotherapy.

## 2. Materials and Methods

### 2.1. Preliminary Study

#### 2.1.1. Patients

 Between December 2010 and January 2013, 625 patients underwent radiotherapy at Ishikawa Prefectural Central Hospital. We prescribed SRT to 5 patients who suffered from lymphedema among them. They had already undergone some treatment for lymphedema or had received no treatment because of its location in the head and neck. We excluded 1 patient whose lymphedema had been caused by an operation. Therefore, we evaluated 4 patients with lymphedema caused by radiotherapy. 

#### 2.1.2. Radiotherapy Planning

 Patients were treated by radiotherapy using 6-MV X-rays from the Novalis Tx image-guided radiotherapy system (Varian, CA, USA, and Brainlab, Feldkirchen, Germany) or 4-MV or 10-MV X-rays from Clinac 21EX (Varian, CA, USA). Stereotactic radiotherapy and intensity-modulated radiotherapy (IMRT) were performed with Novalis Tx, and CLINAC 21EX was used for conventional radiotherapy. Patients were immobilized using a custom-made thermoplastic body cast (Hip-Fix, Med-Tec, Orange City, IA, USA), a Type-S head and shoulder thermoplastic mask (CIVCO, Iowa City, IA, USA), or a customized Vac-Lok (Med-Tec, Orange City, IA, USA) bag that conformed to both the patient's body contours and the treatment box. They were used for head and neck treatment using CLINAC 21EX and for treatment anywhere in the body using Novalis Tx.

#### 2.1.3. Target Localization and Treatment Delivery

 On the treatment day, the patients were again appropriately immobilized on the custom-made thermoplastic cast and Vac-Lok bag box. Initial setup was based on bone anatomy using two-dimensional orthogonal kV images registered to digitally reconstructed radiographs. In the case of IMRT, additional shifts for accurate setting up within the treatment field were performed using three-dimensional cone beam computed tomography (CBCT): 200° gantry rotation, 100 kV, 20 mA, and 20 mS for the head and neck; 200° gantry rotation, 125 kV, 80 mA, and 25 mS for the body. Treatment was then delivered, with the entire process of setting up and treatment taking approximately 15–30 minutes per patient.

#### 2.1.4. Grading of Lymphedema

 The severity of lymphedema was graded using the Common Terminology Criteria for Adverse Events (CTCAE) version 4.0 and the Numerical Rating Scale (NRS). The NRS is a symptom-rating scale from 0 to 10. Zero indicates no symptoms, and 10 indicates the worst symptom that a patient can imagine. Clinical evaluation was performed before and after the administration of SRT.

#### 2.1.5. SRT Application

 SRT (Tsumura Co., Tokyo, Japan) at a dose of 3.0 g was administered orally as a solution three times daily immediately before meals during radiotherapy. None of the patients received diuretic drugs or steroid drugs during the study.

## 3. Results

### 3.1. Preliminary Results in 4 Patients

Table 1 shows the patient characteristics. The mean age of the 4 patients was 64 years (range 33–69). Three of them had a cancer (in the tongue, breast, and mesopharynx, resp.), and one had Kimura disease. Two patients were treated with radiotherapy using Novalis Tx, and the other two cases were treated using CLINAC 21EX. We delivered 2 or 3 Gy per fraction, and the total dose of radiotherapy was 30 to 66 Gy. Three patients had undergone an operation before radiotherapy. They had undergone their last operation 1–15 years ago. Three patients received chemotherapy before radiotherapy and 1 of them received concurrent chemotherapy. Two received SRT after 12 Gy. Two patients received SRT after they finished the total course of radiotherapy (63 or 66 Gy). The compliance was good. Prior to SRT treatment, all patients had grade 2 lymphedema according to the CTCAE, and all patients had symptoms scored as 4 to 10 according to the NRS. After 2 or 3 days of SRT treatment, lymphedema of 2 patients treated with SRT after they received 12 Gy of a score of 10 improved to 0, but lymphedema of the other 2 patients treated with SRT after they finished the entire radiotherapy of a score of 4 or 8 did not improve. Patient 3 decided to stop taking SRT after 3 days because cough and fever appeared. After radiotherapy, in 2 patients who took SRT from the time when they received 12 Gy, lymphedema of a score of 2 improved to a score of 1 according to the CTCAE, but the other two cases showed no change (Table 2).

### 3.2. Case Report

**Figure 1:**
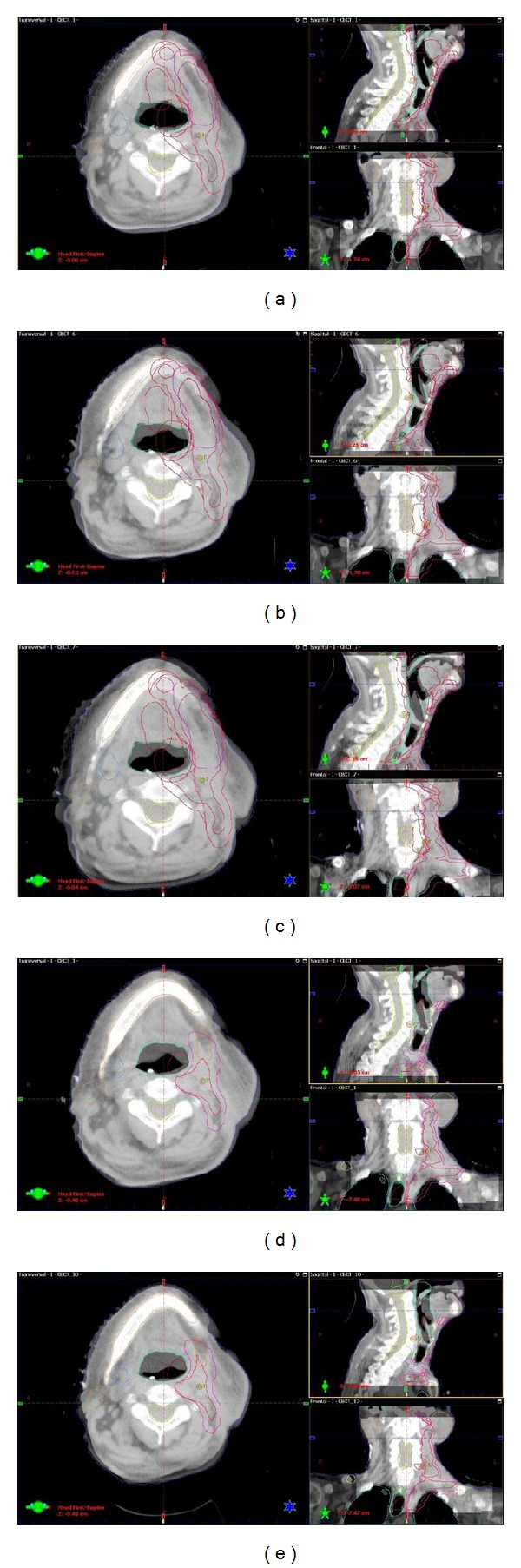
CBCT images of patient 1 on the first day of radiotherapy (a), first day of SRT medication (before medication) (b), 3 days later (c), 14 days later (adaptive radiotherapy) (d), and 28 days later (last day of radiotherapy) (e). Lymphedema improved 3 days later, but it got worse on one occasion. It improved 28 days later.

 A 69-year-old man (patient 1) received radiotherapy using Novalis Tx for cervical lymph node metastases after an operation for tongue cancer. He presented with bleeding from the tongue and visited a local hospital in May 2012. He was referred to Ishikawa Prefectural Central Hospital and was diagnosed with tongue cancer with lymph metastasis (T2N1M0, stage III). He was initially treated by radiotherapy with arterial infusion chemotherapy for only tongue cancer. Next, he was treated by tongue cancer surgery with neck lymph node dissection. Pathologic examination revealed invasion to three regional lymph nodes with extracapsular extension. These findings indicated a high probability of local recurrence and distant metastases and a high risk of death. Therefore, an oral surgeon suggested radiotherapy of the neck region. Two weeks after the start of radiotherapy (12 Gy), lymphedema of the cheek progressed due to the influence of operation and radiotherapy, and the wearing of the custom-made thermoplastic cast became difficult. Therefore, 9.0 g/day of SRT was administered. After 3 days, he demonstrated a dramatic improvement in lymphedema of the cheek and we could easily fit the mask on him.  Figure 1 shows CBCT demonstrating the improvement of lymphedema.

## 4. Discussion

 Lymphedema is a troublesome complication of radiotherapy, especially for patients wearing a custom-made thermoplastic cast. Massive lymphedema leads to temporary discontinuation of radiotherapy because the custom-made thermoplastic cast and treatment planning (adapted plan) need to be made again to ensure accuracy of the treatment. Therefore, it is important to control lymphedema, especially for the patients treated with a custom-made thermoplastic cast. Complications of radiotherapy have decreased with the development of machines and technology for radiotherapy. One of these technological advances is IMRT. The intensity of radiation beams can be changed in IMRT during treatment to spare more adjacent normal tissues than during conventional radiotherapy [15]. Owing to this, an increased dose of radiation can be delivered to the tumor using IMRT. IMRT is a type of conformal radiation, in which radiation beams are shaped to ensure close approximation to the shape of the tumor. When it is applied, improvement in the tumor control rates and reduction of complications can be expected. Nevertheless, some patients suffer from irreversible adverse effects of radiotherapy like lymphedema, for which Western medicine is not useful. In this study, lymphedema in patient 1 progressed during radiotherapy and it became difficult to fit the custom-made thermoplastic cast. Kishida et al. [16] concluded that SRT is useful for the prevention and early recovery of postoperative leg edema after total hip arthroplasty with an association of rapid CRP reduction.

 The mechanism of lymphedema due to radiotherapy has been postulated as follows [17]. The initial fractional doses of irradiation would destroy cells in the vegetative intermitotic cell and differentiating intermitotic cell compartments and reduce the production of cells that normally flow into the postmitotic compartment. The lining or mucous membrane thins and, as the dose builds, the connective tissue becomes edematous; this causes expansion or stenosis of blood vessels. These disorders are typically recovered immediately. However, in the chronic clinical period, cells are sloughed away and fibrosis increases, and blood vessels and lymphatic duct become stenosed and obstructed. These microcirculation disorders cause repeated edema. SRT exhibits pharmacological effects via the following actions. (1) Diuretic actions: SRT shows diuretic actions due to an antagonistic action on the mineralocorticoid receptor, exerted by saikosaponin H, which is a principal component of SRT [13]. (2) Anti-inflammatory actions: there have been several reports about the relationship between SRT and inflammation [13, 18], which are discussed below. Aldosterone, whose function was reportedly suppressed by SRT, produced reactive oxygen species and activated nuclear factor kappa-B (NF-*κ*B) [19, 20]. NF-*κ*B activation leads to increased expression of chemokine and proinflammatory cytokine genes, including tumor necrosis factor-*α*, interleukin-6, inducible nitric oxide synthase, and vascular endothelial growth factor [21–24]. In addition, saikosaponin inhibited T-cell activation via the suppression of NF-*κ*B [13]. Thus, both direct and indirect actions of suppression of NF-*κ*B activity might have contributed to a change in the normalization of CRP early in the study. Oral administration of SRT to rats increased the plasma ACTH level and the expression of proopiomelanocortin (an ACTH precursor in the anterior pituitary lobe) mRNA [25]. Furthermore, these actions were inhibited by antiserum corticotropin-releasing factor [26].

 SRT has not been investigated (drug use investigations, etc.) to determine the incidence of adverse reactions. There have been several reports of adverse reactions including interstitial pneumonitis, pseudoaldosteronism, myopathy, fulminant hepatitis, hepatic dysfunction, and jaundice [27]. In this study, one patient decided to stop taking SRT after 3 days because cough and fever appeared. Fever, cough, dyspnea, abnormal pulmonary sound (fine crackle), and ground-glass-like and reticular opacities predominantly in the bilateral lower lung fields on chest computed tomography were observed when interstitial pneumonitis occurred. The pathogenesis of drug-induced pneumonitis is not completely understood; intolerance to side effects, secondary effects, and idiosyncratic allergic reactions is thought to be the most important factor [28]. Pneumonitis generally develops 1-2 weeks after the start of administration, and bronchoalveolar lavage and histologic examination of lung biopsies reveal the features of eosinophilic pneumonitis [29, 30]. Allergic pneumonitis is particularly mediated via types III and IV allergic reactions [31]. The patient who stopped taking SRT after 3 days visited our hospital 1 month later, so it was too late to examine the cause of her disease and it was too early for interstitial pneumonitis by SRT to have developed. The Japanese Ministry of Health and Welfare had approved 148 types of Kampo drug by 1999. Nakagawa et al. [32] conducted national surveillance of drug-related pneumonitis in Japan and reported that cases of Kampo drug-related pneumonitis accounted for as much as 10% (75 cases due to 13 kinds of Kampo drug) of all cases of drug-related pneumonitis in Japan from 1984 to 1996. Using their data, 84% of cases of Kampo drug-related pneumonitis were associated with drugs containing Ougon (*Scutellaria* root, dried root of *Scutellaria baicalensis* Georgi), that is, 49 cases for Shosaikoto, 9 for saibokuto, 4 for SRT, 1 for scihaito, 1 for daisaikoto, 1 for saikokeishito, and 1 for saikokeishikanshouto. Including these drugs, the Japanese Ministry of Health and Welfare has approved 29 kinds of Kampo drug that contain Ougon. Ougon is thought to be the most important cause of pneumonitis because of previously reported highly positive lymphocyte stimulation tests [33].

 Tsumura & Co. investigated reports on Shosaikoto drug use from October 1995 to March 1997, including abnormal laboratory test results, and 88 adverse reactions were observed in 69 of 2,495 patients (2.8%). The incidence of interstitial pneumonitis was less than 0.1% [34]. Nakagawa et al. [32] showed that the rate of interstitial pneumonitis as an adverse reaction of SRT was lower than the rate for Shosaikoto. Therefore, it is rare for interstitial pneumonitis to develop as an adverse reaction of SRT.

## 5. Conclusions

 It is important to control lymphedema caused by radiotherapy because it leads to improvement of the tumor control rate. SRT can easily be taken continuously for a long period with few side effects. SRT also has pharmacologic action in patients with lymphedema caused by radiation. In this study, we investigated the effectiveness of SRT in only 4 cases, so further research with much more patients and, preferably, prospective randomized studies are needed to confirm our findings.

## Figures and Tables

**Table 1:** Patient characteristics.

Case	1	2	3	4
Age/sex	69/M	33/M	68/F	60/M
Performance status	2	0	0	0
Area for radiotherapy	Neck	Neck	Axilla	Neck
Primary lesion	Tongue cancer	Kimura disease	Breast cancer	Mesopharyngeal carcinoma
Total dose (Gy)	50	30	63	66
Machine for radiation therapy	Novalis Tx	CLINAC 21EX	Novalis Tx	CLINAC 21EX
Type of radiation therapy	IMRT	Conventional	IMRT	Conventional
Past operations	Yes	Yes	Yes	No
Chemotherapy	Yes	No	Yes	Yes
Past	Yes	No	Yes	Yes
Concurrent	No	No	No	Yes

Total dose at start of medication (Gy)	12	12	63	66

**Table 2:** Scores for lymphedema before and after medication.

Case	1	2	3	4
Total dose at start of medication (Gy)	12	12	63	66
NRS				
Before	10	10	8	4
After	0	0	8	4
CTCAE				
Before	2	2	2	2
After	1	1	2	2
Results	Improved	Improved	Not changed	Not changed

NRS: Numerical Rating Scale; CTCAE: Common Terminology Criteria for Adverse Events.
